# Genetic analysis of potential biomarkers and therapeutic targets in ferroptosis from psoriasis

**DOI:** 10.3389/fimmu.2022.1104462

**Published:** 2023-01-04

**Authors:** Man-Ning Wu, Dong-Mei Zhou, Chun-Yan Jiang, Wei-Wen Chen, Jia-Chi Chen, Yue-Min Zou, Tao Han, Li-Jia-Ming Zhou

**Affiliations:** Department of Dermatology, Beijing Hospital of Traditional Chinese Medicine, Capital Medical University, Beijing, China

**Keywords:** bioinformatics, psoriasis, diagnostic, ferroptosis, immune microenvironment

## Abstract

**Introduction:**

Ferroptosis is associated with multiple pathophysiological processes. Inhibition of ferroptosis has received much concern for some diseases. Nonetheless, there is no study comprehensively illustrating functions of ferroptosis-related genes (FRGs) in psoriasis.

**Methods:**

In this study, FRGs together with psoriasis-associated data were obtained in Ferroptosis Database (FerrDb) and gene expression omnibus (GEO) database separately. This work identified altogether 199 psoriasis-associated DE-FRGs, and they were tightly associated with immunity and autophagy modulation. Thereafter, the present study utilized SVM-RFE and LASSO algorithms to identify NR5A2, CISD1, GCLC, PRKAA2, TRIB2, ABCC5, ACSF2, TIMM9, DCAF7, PEBP1, and MDM2 from those 199 DE-FRGs to be marker genes. As revealed by later functional annotation, the marker genes possibly had important effects on psoriasis through being involved in diverse psoriasis pathogenesis-related pathways such as cell cycle, toll-like receptor (TLR), chemokine, and nod-like receptor (NLR) pathways. Moreover, altogether 37 drugs that targeted 11 marker genes were acquired. Besides, based on CIBERSORT analysis, alterations of immune microenvironment in psoriasis cases were possibly associated with PRKAA2, PEBP1, CISD1, and ACSF2.

**Discussion:**

Taken together, this work established the diagnostic potency and shed more lights on psoriasis-related mechanism. More investigations are warranted to validate its value in diagnosing psoriasis before it is applied in clinic.

## 1 Introduction

Psoriasis, the frequently seen, chronic autoimmune skin disorder, shows the features of epidermis modification due to keratinocyte hyperproliferation, excess immunocyte infiltration, together with inflammatory cytokine accumulation (1). In clinical practice, psoriasis has a global incidence rate of 2-3% (2). Nonetheless, its pathogenesis remains largely unclear. Numerous factors such as environmental and genetic factors are suggested to enhance psoriasis progression (3). Aberrant infiltrating immunocyte-activated keratinocyte interaction can induce psoriatic skin inflammation. IL-17 axis and immunocytes are related to the psoriasis pathogenesis (4). Keratinocytes have been identified in previous studies to be initiators for inflammation, which are important for amplifying inflammatory cascade by secreting cytokines and chemokines (5–7). The internal changes in epidermal keratinocytes have induced higher susceptibility to external triggers, thus facilitating the inflammatory process.

Ferroptosis represents the iron-dependent programmed cell death type that is proposed in 2012, and it is distinct from necrosis, apoptosis, autophagy and pyroptosis (8). Ferroptosis shows the features of mitochondrial atrophy, elevated mitochondrial membrane density, involvement of specific genes, along with iron and lipid reactive oxygen species (L-ROS) accumulation (9, 10). Typically, lipid radical formation, lipid peroxidase 4 (GPX4) inactivation and glutathione (GSH) deletion can catalyze iron biochemical metabolism (11). The iron level in circulation has an essential effect on ferroptosis occurrence. Iron chelating agent application helps to suppress Erastin-induced ferroptosis, and transferrin expression onto cell membrane can enhance cell sensitivity to ferroptosis (8). Through the release of damage-associated molecular patterns (DAMPs) and alarmins, ferroptosis can cause not only cell death but also inflammatory reactions (12). Psoriatic keratinocytes possess an enhanced ability to resist apoptosis (13), while they are more susceptible to necroptosis (14). According to several reports, necroptosis triggers psoriatic inflammation in keratinocytes by releasing DAMPs and activating inflammasomes. It cannot be ignored that IMQ-induced psoriasis-like dermatitis can be effectively treated with Fer-1 by inhibiting ferroptosis.

Oxidative stress (OS) has been recently suggested to be tightly associated with ferroptosis, while ferroptosis can be activated by excess ROS generation (15). Relation of ferroptosis with psoriasis remains largely unclear, but OS in psoriasis is suggested to cause abnormalities in the ferroptosis-related pathways (16, 17). To take an example, epidermal GPX4 deletion promotes cyclooxygenase-2 expression and lipid peroxidation within the entire skin, thus inducing epidermal hyperplasia and dermal inflammatory infiltration within perinatal mice. Besides, GPX4 deletion decreases keratinocyte adhesion into the culture while increasing intracellular lipid peroxidation degree (18). As reported in one paper, the important ferroptosis regulator GPX4 shows decreased expression in psoriatic skin lesions compared with unaffected skin and samples from normal controls (NCs). Meanwhile, the cellular import of iron was increased, suggesting that there is activation of ferroptosis in psoriatic skin lesions (19). At present, ferroptosis inhibitors are identified to resist inflammation in the acute kidney injury, neurodegenerative disorder and intracerebral hemorrhage experimental models (20–22). Therefore, more studies are needed to investigate whether ferroptosis is involved in the psoriasis pathogenesis. Consequently, this work aimed to examine whether Ferroptosis-related genes could be the accurate biomarkers for psoriasis and analyze their effects on immune microenvironment by bioinformatics analysis.

## 2 Materials and methods

### 2.1 Data source

The present work acquired gene expression profiles in psoriasis and healthy samples in GEO database. GSE117239 dataset included altogether 324 samples, with 240 psoriasis and 84 healthy samples, which served as the training set in our analysis. GSE13355 dataset contained 58 psoriasis and 64 healthy samples, which served as validation set for verifying marker gene levels. In addition, FRGs (n=322) adopted in the present work were acquired based on FerrDb. **Table S1** displays more details of genes. Drugs that targeted marker genes were predicted using Drug Gene Interaction Database (DGIdb).

### 2.2 Differentially expressed genes identification

Expression profiles for 199 FRGs in psoriasis and healthy samples obtained based on GSE117239 database were collected. Later, differentially expressed FRGs (DE-FRGs) were detected between two groups by Wilcoxon rank-sum test in R with p<0.05 being set as the significance level. And adjust P-values by Benjamini-Hochberg correction for multiple testing (**Table S2**).

### 2.3 Functional annotation

To further analyze functions of DE-FRGs, Gene Ontology (GO) together with Kyoto Encyclopedia of Genes and Genomes (KEGG) enrichment was conducted on these genes by R software “clusterProfiler” package (V4.4.4).

### 2.4 Best gene biomarkers for the diagnosis of psoriasis

Using glmnet package, this work adopted the least absolute shrinkage and selection operator (LASSO) algorithm for reducing data dimension (23, 24). DE‐FRGs in psoriasis versus healthy samples were preserved to select features, while LASSO algorithm was employed to identify psoriasis-related gene biomarkers. At the same time, this work utilized SVM package to construct the support vector machine‐recursive feature elimination (SVM‐RFE) model, and mean misjudgment rates were used for comparison with 10‐fold cross‐validations (25). Additionally, overlapped biomarkers obtained by both algorithms were deemed as the best gene biomarkers for psoriasis. Receiver operating characteristic (ROC) curves were plotted, and area under the curve (AUC) values, sensitivity, specificity and accuracy were determined to evaluate whether our selected gene markers were of diagnostic value. Moreover, by using R package glm, a seven marker genes-based logistic regression model was built for predicting GSE117239 dataset sample types. Likewise, ROC curves were used to evaluate whether our constructed logistic regression model was of diagnostic value.

### 2.5 Single‐gene gene set enrichment analysis

The R software GSEA (V.4.1.0) package was adopted for ssGSEA. For exploring pathways enriched by those seven marker genes, associations of marker genes with the remaining genes were analyzed based on GSE117239 dataset. Thereafter, each gene was sorted based on the high-to-low correlations, while those sorted genes were listed into the gene set for analysis. Further, this work deemed the KEGG pathway set as the predefined set for detecting enrichment levels in the gene set. Specific enrichment results of each marker gene were integrated into **Table S3**.

### 2.6 Single‐gene gene set variation analysis

R software GSVA (V.1.38.0) package was utilized for GSVA (26). The present work adopted KEGG pathway set to be background gene set for GSVA of diverse marker genes. Meanwhile, limma package was utilized for analyzing different GSVA scores for marker genes in high- and low-expression groups upon the significance levels of |t| >2 and p<0.05. The pathway was activated into high‐expression group for t>0; otherwise, it was activated into low-risk group.

### 2.7 Immune infiltration analysis

CIBERSORT, an approach for characterizing cellular composition in complicated tissues based on gene expression data (27). The present work estimated 22 infiltrating immune cell types in GSE117239 dataset samples with CIBERSORT software (**Table S4**). Besides, CIBERSORT was also used to predict the immune genes and the immune functions in each tissue.

### 2.8 Statistical analysis

Wilcoxon rank-sum test was adopted to compare 2 groups. Relations of 199 DE‐FRGs were analyzed by Pearson correlation analysis. Jvenn package was employed for Venn diagram plotting. Statistical analysis was completed with R software.

### 2.9 Validation in a single cell dataset

This work obtained scRNA-seq count matrix based on GSE162183 dataset. This dataset covered 6 samples, with 3 psoriasis and 3 normal samples, and the 3 psoriasis tissues (GSM4946164, GSM4946165, GSM4946166) were selected for analysis. Quality control (QC) was conducted with Seurat R package (28). The cell removal criteria were as follows, (a) RNA counts <50 and (b) mitochondrial gene expression ratio >5%. NormalizeData function in Seurat was utilized to normalize data. We later selected 14 most significant principal components as well as 1500 most significant variable genes in later analysis. Thereafter, Seurat’s FindClusters function (resolution = 0.5) was used to detect cell clusters, while 2D t-distributed stochastic neighbor embedding (tSNE) was adopted for display (29). By adopting SingleR package, this study compared cells in diverse clusters to annotated reference dataset (30). Cluster annotation was completed based on those identified cell markers and comparison results.

## 3 Results

### 3.1 DE‐FRGs identification from GSE117239 database

**Figure 1:**
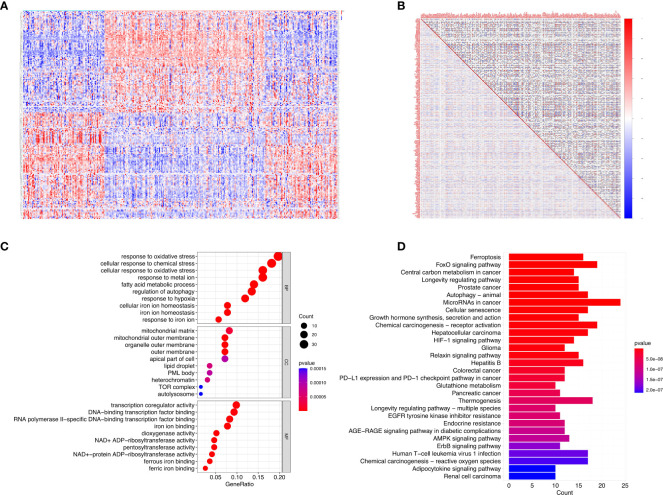
DE-FRGs expression levels in psoriasis and functional analyses for the DE-FRGs. **(A)** Violin plots show expression patterns of DE-FRGs across samples. **(B)** The correlation of these genes. **(C)** GO enrichment analyses indicated that DE-FRGs were significantly related to the function of ‘response to oxidative stress’, ‘mitochondrial matrix’ and ‘transcription coregulator activity’. **(D)** Reactome pathway analyses indicated that the MicroRNAs in cancer, FoxO signaling pathway, and Chemical carcinogenesis−receptor activation were enriched.

There were 199 DE-FRGs identified from 322 FRGs in psoriasis versus healthy samples in GSE117239 dataset, which included 99 with up-regulation whereas 100 with down-regulation. **Figure 1A** displays the clustering heatmap for DE‐FRGs expression profiles in diverse samples, and **Figure 1B** exhibits gene associations.

### 3.2 Functional annotation of DE‐FRGs

For elucidating DE‐FRGs-associated biological functions as well as pathways, GO functional annotation together with Reactome pathway analysis was carried out. Therefore, GO-molecular function (MF) annotation revealed the significant enrichment of DE‐FRGs into “transcription coregulator activity”, “RNA polymerase II specific DNA-binding transcription factor binding”, and “DNA-binding transcription factor binding” (**Figure 1C**). With regard to cellular component (CC), DE‐FRGs were markedly associated with “mitochondrial matrix”, “organelle outer membrane”, and “mitochondrial outer membrane”. In addition, GO-biological process (BP) annotation suggested the close relation of DE‐FRGs with “response to oxidative stress”, “cellular response to oxidative stress”, and “cellular response to chemical stress”. Based on Reactome pathway analysis, MicroRNAs in cancer, FoxO signaling pathway and Chemical carcinogenesis-receptor activation were remarkably enriched (**Figure 1D**). According to the above results, DE‐FRGs might have critical effects on psoriasis pathogenesis through regulating autophagy, cytokines, kinases and immune cells.

### 3.3 11 DE‐FRGs served as genes to diagnose psoriasis

**Figure 2:**
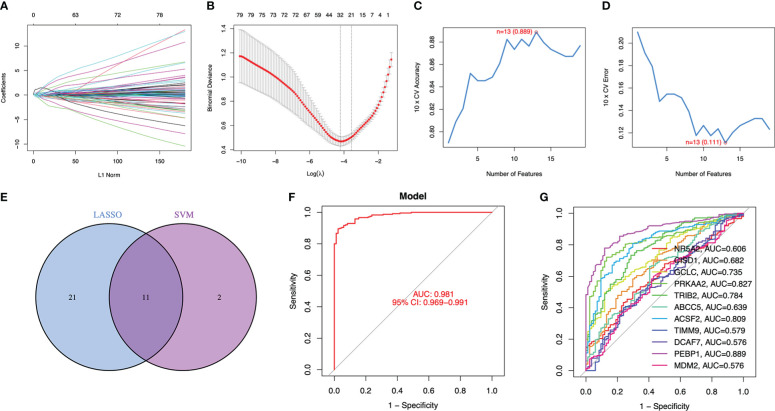
11 DE-FRGs were identified as diagnostic genes for psoriasis. **(A, B)** By LASSO logistic regression algorithm, with penalty parameter tuning conducted by 10-fold cross-validation, was used to select 32 psoriasis-related features. **(C, D)** SVM-RFE algorithm to filter the 13 DE-FRGs to identify the optimal combination of feature genes. Finally, 11 genes (maximal accuracy = 0.889, minimal RMSE = 0.111) were identified as the optimal feature genes. **(E)** The marker genes obtained from the LASSO and SVM-RFE models. **(F)** Logistic regression model to identify the AUC of disease samples. **(G)** ROC curves for the 11 marker genes.

To consider changes in psoriasis cases compared with normal subjects, this work focused on predicting whether DE-FRGs could be used in disease diagnosis. Subsequently, 2 different machine learning algorithms LASSO and SVM-RFE were adopted for analysis based on GSE117239 dataset, for the sake of screening DE‐FRGs significantly distinguishing psoriasis from healthy subjects. The penalty parameter was tuned by 10-fold cross-validation in LASSO logistic regression, which selected 32 psoriasis‐related features (**Figures 2A, B**). Afterwards, SVM‐RFE algorithm was applied in filtering 13 DE‐FRGs for identifying the best feature gene combination. At last, this work detected 11 genes (minimal RMSE =0.111, maximal accuracy =0.889) to be best feature genes (**Figures 2C, D**). Thereafter, marker genes acquired based on the above two algorithms were intersected to obtain 11 marker genes (NR5A2, CISD1, GCLC, PRKAA2, TRIB2, ABCC5, ACSF2, TIMM9, DCAF7, PEBP1, MDM2) in subsequent analyses (**Figure 2E**).

Using R package glm, these 11 marker genes identified were used to build the logistic regression model. According to later ROC curve analysis, the logistic regression model built based on these 11 marker genes well distinguished psoriasis from healthy samples, and the AUC value was 0.981 (**Figure 2F**). Besides, for elucidating whether single genes could be used to differentiate psoriasis from healthy controls, this work plotted ROC curves for those 11 marker genes. According to **Figure 2G**, AUC value was >0.57 of these 11 genes. Consequently, our logistic regression model was accurate and specific in distinguishing psoriasis from healthy samples compared with single marker genes.

### 3.4 Marker genes showed tight relation with various psoriasis‐related pathways

For better exploring the possible roles of marker genes in distinguishing psoriasis from healthy samples, the single‐gene GSEA‐KEGG pathway analysis was carried out. **Figure S1** displays those 6 pathways associated with marker genes. Marker genes were comprehensively analyzed, as a result, they were found to be significantly associated with cell cycle, cytokine cytokine receptor interaction, chemokine pathway, nod like receptor pathway, jak stat pathway, rig-i-like receptor pathway, toll like receptor pathway, together with different disease pathways (graft versus host disease, prion disease, and type I diabetes mellitus). Furthermore, markers genes were significantly related to Adherens junction, Pathway in cancer, TGF BETA pathway, Regulation of actin cytoskeleton, PPAR pathway, Allograft rejection, Hematopoietic cell lineage, Aminoacyl TRNA biosynthesis, Intestinal immune network for IgA production, and Steroid biosynthesis as well. Besides, ACSF2, MDM2 and TRIB2 were tightly associated with the ‘jak stat pathway’. All marker genes, except for ABCC5 and DCAF7, were all related to ‘nod like receptor pathway’.

Thereafter, this work analyzed pathways with differential activation levels in psoriasis compared with normal groups according to GSVA and marker gene expression levels. Consequently, NR5A2 down-regulation might cause psoriasis *via* the activation of ‘MATURITY ONSET DIABETES OF THE YOUNG’, while its overexpression activated ‘FOLATE BIOSYNTHESIS’, ‘NOD LIKE RECEPTOR SIGNALING PATHWAY’, and ‘EPITHELIAL CELL SIGNALING IN HELICOBACTER PYLORI INFECTION’ (**Figure S2A**). Besides, CISD1 overexpression was found to activate ‘CIRCADIAN RHYTHM MAMMAL’, and its down-regulation was associated with ‘PYRIMIDINE METABOLISM’ (**Figure S1B**). In addition, GCLC down-regulation was associated with ‘FOLATE_BIOSYNTHESIS’, and its up-regulation led to activation of ‘COMPLEMENT AND COAGULATION CASCADES’ (**Figure S2C**). ACSF2 up-regulation was found to activate numerous psoriasis‐related pathways, including RIG-I LIKE RECEPTOR, TOLL LIKE RECEPTOR, NOD LIKE RECEPTOR pathways (**Figure S2D**). Notably, TRIB2 up-regulation was directly associated with ‘TAURINE AND HYPOTAURINE METABOLISM’. In the TRIB2 low‐expression group, ‘CYTOKINE CYTOKINE RECEPTOR INTERACTION’ and ‘TOLL LIKE RECEPTOR SIGNALING PATHWAY’ were significantly enriched (**Figure S2E**). Moreover, PEBP1 up-regulation was associated with various psoriasis pathogenesis-related pathways, like ‘NOD LIKE RECEPTOR SIGNALING PATHWAY’, ‘JAK‐STAT SIGNALLING PATHWAY’, ‘CYTOKINE CYTOKINE RECEPTOR INTERACTION’, and ‘TOLL LIKE RECEPTOR SIGNALING PATHWAY’ (**Figure S2K**).

### 3.5 The immune microenvironment of psoriasis tissue

**Figure 3:**
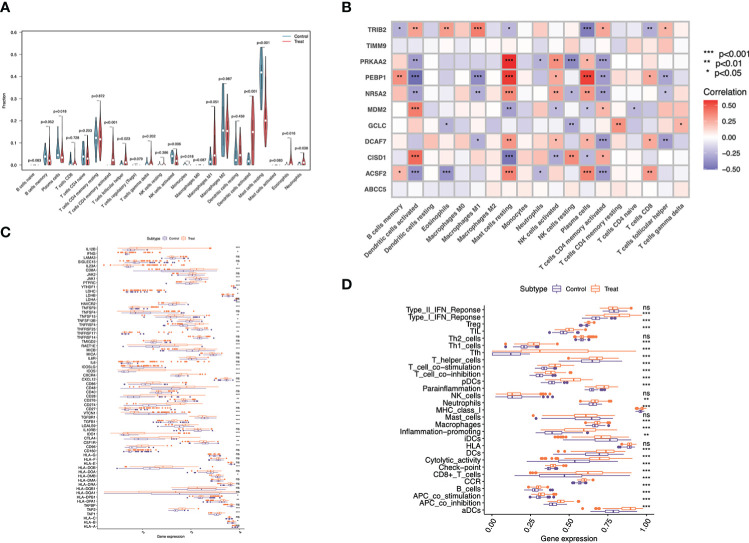
Immune landscape analysis. **(A)** Implemented the CIBERSORT algorithm to explore the differences in the immune microenvironment between psoriasis patients and normal samples. **(B)** Pearson correlation analysis (*p < 0.05, **p < 0.01,*** p < 0.001). **(C)** Immune gene expression and **(D)** immune function in psoriasis compared with normal tissue.

According to our above-mentioned analysis, marker genes showed tight association with immunity. In the meantime, evidence supported that psoriasis was closely related to the immune microenvironment. Consequently, CIBERSORT algorithm was utilized for exploring different immune microenvironment in psoriasis cases relative to healthy controls. According to **Figure 3A**, Mast cells resting exhibited a decreased proportion in psoriasis compared with healthy samples, whereas plasma cells, T cells follicular helper, T cells CD4 memory activated, monocytes, NK cells activated, Dendritic cells activated, Neutrophils and Eosinophils had higher proportions in psoriasis samples.

As suggested by Pearson correlation analysis, Mast cells resting were significantly positively related to PRKAA2, PEBP1, NR5A2 and ACSF2 respectively, and strongly negatively correlated with CISD1. Plasma cells were strongly positively related to PEBP1 and ACSF2, and negatively correlated with TRIB2. Dendritic cells activated were significantly positively related to CISD1 and MDM2, but markedly negatively correlated with ACSF2 and PEBP1. Additionally, T cells CD4 memory activated had strong negative correlations with PRKAA2, DCAF7 and ACSF2. ACSF2 was also negatively correlated with Eosinophils, and PRKAA2 was negatively correlated with NK cells resting (**Figure 3B**).

For investigating different immune profiles in psoriasis and normal samples, different immune-related genes (IRGs) and immune-related functions were compared. As a result, immunostimulatory genes showed higher expression levels within psoriasis than normal tissues, such as CD86, CD48, TNFRSF4, TNFRSF25, ICOS, and CXCR4. By contrast, some immunosuppressive genes like VTCN1 and TGFBR1, were up-regulated in normal cases. A variety of immune functions in psoriasis compared with normal samples were compared, as a result, Inflammation promoting, cytolytic activity, T cells and B cells functions might be more potent among psoriasis samples, thereby suggesting the immune active status of psoriasis samples (**Figure 3C**). Different immune functions in psoriasis compared with normal samples were compared. Most of immune functions were significantly enriched in psoriasis relative to normal samples, such as ‘B cells’, ‘Macrophages’, ‘human leukocyte antigen (HLA)’ and ‘inflammation promoting’. Such results suggested that psoriasis patients exhibited increased and complex immune components (**Figure 3D**).

### 3.6 Marker gene‐targeted drugs prediction

**Figure 4:**
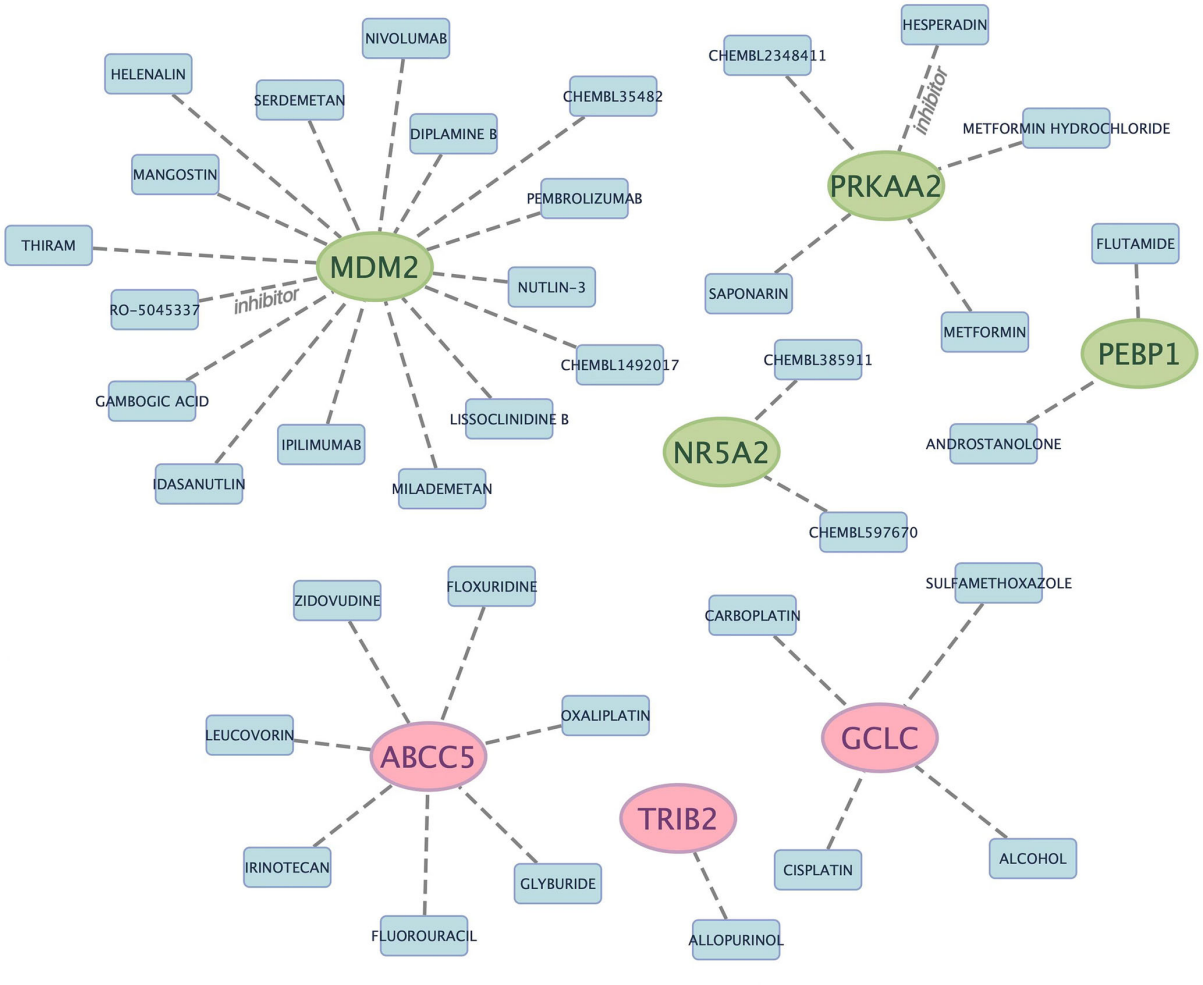
Prediction of marker gene-targeted drugs. The drugs may target marker genes through the DGIdb database and the interaction relationship between the two.

To identify drugs possibly targeting marker genes, DGIdb database was adopted for analysis, with tow-parameter interaction relation being set at defaults (**Table S5**). **Figure 4** displays Cytoscape software-based result visualization. Altogether 37 drugs that targeted marker genes were queried, which included 16 for MDM2, 7 for ABCC5, 5 for PRKAA2, 4 for GCLC, 2 for NR5A2, 2 for PEBP1, and 1 for TRIB2. However, drugs targeting CISD1, ACSF2, TIMM9 and DCAF7 genes were not predicted. HESPERADIN was an inhibitor of PRKAA2, while RO-5045337 was an inhibitor of MDM2.

### 3.7 Marker gene expression levels in validation set

**Figure 5:**
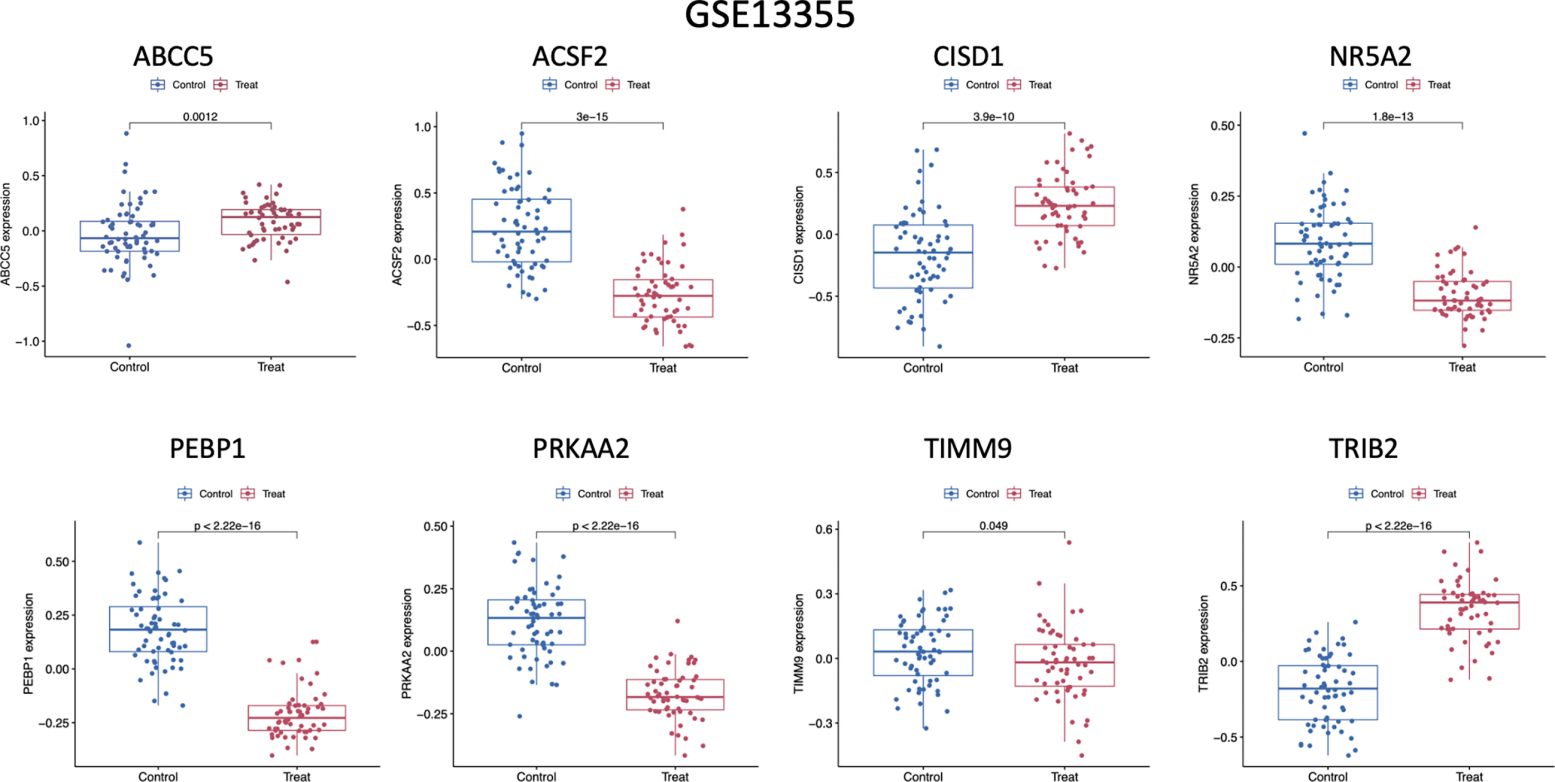
Expression of the marker gene in the validation set. The expression of marker genes in the GSE13355 dataset.

At last, marker genes expression levels were validated based on GSE13355 dataset. According to our results, NR5A2, CISD1, PRKAA2, TRIB2, ABCC5, ACSF2, TIMM9 and PEBP1 showed similar expression profiles to those in GSE117239 dataset. Typically, CISD1 (p = 3.9e-10), TRIB2 (p < 2.22e−16), and ABCC5 (p=0.0012) levels among psoriasis cases increased compared with healthy controls, whereas PRKAA2 (p < 2.22e−16), ACSF2 (p = 3e−15), TIMM9 (p= 0.049), PEBP1 (p < 2.22e−16) and NR5A2 (p=1.8e−13) levels decreased among psoriasis cases (**Figure 5**).

### 3.8 Validation in a single-cell dataset

**Figure 6:**
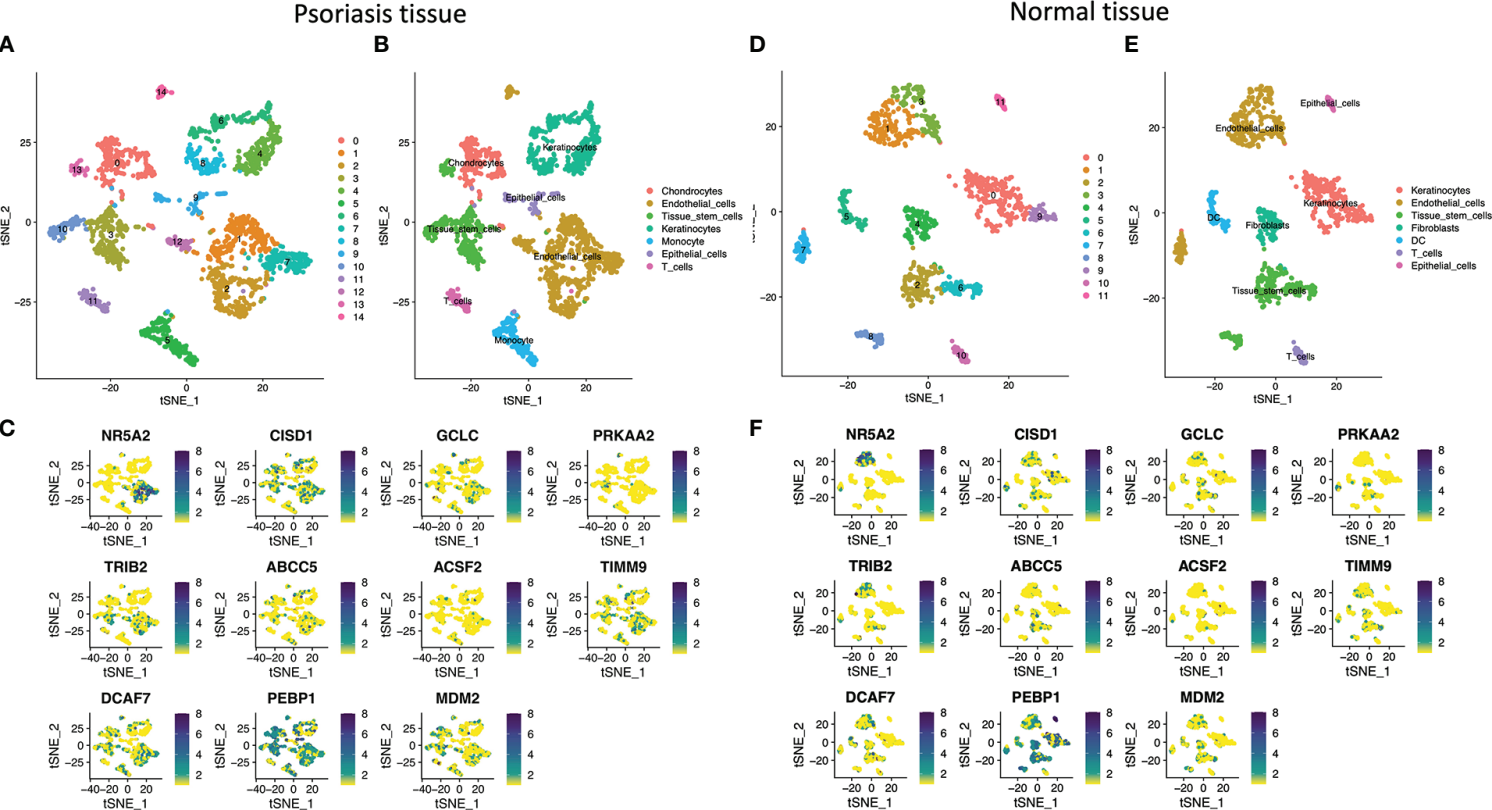
Cell-type classification in psoriasis and normal tissue. **(A)** and **(D)** t-SNE plot of 14 cell clusters in psoriasis tissue and healthy tissue. **(B)** and **(E)** t-SNE plot exhibiting the cell types in psoriasis. **(C)** and **(F)** t-SNE plot of the marker genes.

This work recruited six psoriasis together with matched healthy samples. Altogether 39,938 cells conformed to QC, including 19969 in psoriasis samples, whereas the remaining in healthy samples. The cells were later classified as 14 clusters (**Figure 6A**). There were 7 main cell types discovered from psoriasis, including Chondrocytes, Endothelial cells, Tissue stem cells, Keratinocytes, Monocyte, Epithelial cells, and T cells (**Figure 6B**). Subsequently, 11 marker genes were analyzed, which were NR5A2, CISD1, GCLC, PRKAA2, TRIB2, ABCC5, ACSF2, TIMM9, DCAF7, PEBP1 and MDM2. These gene levels were later marked in respective cell types (**Figures 6C**). The results of normal tissue were shown in (**Figures 6D–F**). Marker genes within psoriasis samples showed major distribution within Endothelial cells. NR5A2, CISD1, TIMM9, TRIB2 and PEBP1 were marker genes of Endothelial cells. In addition, PEBP1, CISD1 and MDM2 were predominantly expressed in Chondrocytes and Tissue stem cells. PEBP1 was highly expressed in Keratinocytes. For immune cells, T cells and Monocytes were related to PEBP1, MDM2, TIMM9 and DCAF7 in psoriasis group. Marker genes within healthy samples showed major distribution in Endothelial cells. NR5A2 and PEBP1 were marker genes of Endothelial cells.

## 4 Discussion

Recently, an increasing number of articles demonstrate that OS is tightly associated with ferroptosis (8, 17). Ferroptosis represents the iron-dependent, lipid peroxidation-mediated cell death pattern. It is related to different *in-vivo* pathophysiological processes, like neuropathy, ischemia/reperfusion injury and tumor immunity. Ferroptosis is closely related to inflammation within psoriatic lesions. Aberrant lipid metabolism and expression can be detected among psoriasis patients, in particular within keratinocytes collected in psoriatic lesions (31). Lipid oxidation pathway is markedly activated within keratinocytes from psoriasis at the single-cell level, while lipid peroxidation will be promoted in the case of psoriasis (17). The ferroptosis-related cell death is activated within psoriatic lesions as well. For instance, GPX4 shows high expression within various epidermis layers from healthy samples, but low expression within psoriatic skin (17). Moreover, transferrin receptor (TFRC) displays remarkable up-regulation within psoriatic tissues, whereas ferritin light chain (FTL) and ferritin heavy chain 1 (FTH1) exhibit down-regulation (17). FTL and FTH1 are related to iron storage, entry and homeostasis. Selenium level decreases, which is associated with psoriasis severity among cases experiencing long-duration of disease. Furthermore, selenium deficiency is found to impact GPX4 biosynthesis, and this can account for the reduced ferroptosis vulnerability and antioxidant ability among psoriatic cases (32, 33). Nevertheless, little is known about whether ferroptosis is significant in psoriasis. Consequently, the present work focused on identifying possible FRGs in psoriasis based on bioinformatics analysis. We chose numerous appropriate gene chips and used diverse genes and microarray data, which reduced the error rate increased our result reliability, thereby offering the significant clinical reference for prevent and treat psoriasis.

The present work selected altogether 11 DE-FRGs, namely, NR5A2, CISD1,GCLC,PRKAA2,TRIB2,ABCC5,ACSF2,TIMM9,DCAF7,PEBP1,MDM2. For those 11 marker genes, their AUC values were >0.57, demonstrating that they were accurate and specific in differentiating psoriasis cases from healthy samples. Notably, PEBP1, PRKAA2, and ACSF2 had the highest AUC values. PEBP1, the scaffold protein inhibitor of protein kinase cascades, is found to form complexes with 15LO1 and 15LO2 (the 15LO isoforms), while changing the corresponding substrate competence for generating hydroperoxy-PE. Insufficient hydroperoxy-PE reduction because of GPX4 dysfunction or deletion may cause ferroptosis (34). In addition, PRKAA2, a gene responsible for encoding AMPKα2, is suggested to regulate several gene levels related to late myogenesis and differentiation (35). PRKAA2 can suppress cell growth *via* p53 pathway as an AMPK subunit, which thus induces ferroptosis by inhibiting SLC7A11 transcription (36–38). On the other hand, acyl-CoA synthetase family member 2, the ACSF2 product, is related to initial fatty acid metabolism through the catalysis of thioesterification into CoA, as a result, it can participate in multiple anabolic and catabolic pathways. Impairment of acyl-CoA synthetases is reported to be related to metabolic syndrome and insulin resistance (39, 40). Nevertheless, little is known about ACSF2’s function in executing ferroptosis.

Subsequently, single‐gene GSEA‐KEGG pathway analysis was performed to explore potential functions of marker genes. It was found that nod-like receptors (NLRs), toll-like receptors (TLRs) and RIG-I-like receptors (RLRs) were closely related to marker genes. TLRs are the transmembrane receptors detected on intracellular membranes and cell surface (lysosomal wall and endoplasmic reticulum) (41). RLRs and NLRs are the intracellular receptors. After stimulation, receptors can activate various related pathways to increase generation of pro-inflammatory molecules like interferon I (IFN-I) and proinflammatory cytokines like interleukin-1 (IL-1), IL-6, and tumor necrosis factor (TNF). Molecules produced during the process increase the immune cell reflex and activate the nonspecific response system (42, 43).

Furthermore, this work also analyzed the immune cells in psoriasis compared with healthy samples. Compared with normal samples, activated memory CD4+ T cells, Plasma cells, activated NK cells, follicular helper T cells, monocytes, activated dendritic cells, Eosinophils and neutrophils showed higher infiltration levels, while memory resting mast cells displayed a lower infiltration level. Infiltration levels of the above immune cells mostly conformed to prior studies (44). In addition, immune genes and immune function analysis also reported up-regulated gene levels in psoriasis group, which demonstrated that immune expression was aggravated in psoriatic tissues. In our analysis, it was found that PRKAA2 was closely related to ferroptosis and psoriasis, and metformin stimulated the expression of PRAKK2. A significant metformin-genetic variants interaction is reported among genes that encode other proteins related to AMP-activated protein kinase, such as PRAKK2 (45). Previous study has also demonstrated that metformin administration increases p-ERK1/2 and p-AMPK levels within HaCaT cells, but remarkably suppresses human keratinocyte growth by activating MAPK pathway. Our analysis results were consistent with previous studies.

Meanwhile, marker gene levels were validated based on GSE13355 dataset. According to our results, ABCC5, CISD1, TRIB2, NR5A2, PEBP1 and PRKAA2 expression trends conformed to those in GSE117239 dataset. Typically, ABCC5 (p = 0.0012), CISD1 (p= 3.9e−10) and TRIB2 (p < 2.22e−16) levels in psoriasis cases increased compared with healthy samples, whereas NR5A2 (p = 1.8e−13), PEBP1 (p < 2.22e−16) and PRKAA2 (p < 2.22e−16) levels decreased in psoriasis samples.

In line with our aforementioned bioinformatics analysis, those 11 DE-FRGs expression was assessed based on GSE13355 dataset. Consequently, the gene levels increased in psoriasis tissues but decreased in healthy samples, consistent with the results obtained from GSE117239 dataset.

## Conclusion

In conclusion, NR5A2, CISD1, GCLC, PRKAA2, TRIB2, ABCC5, ACSF2, TIMM9, DCAF7, PEBP1, and MDM2 are identified as marker genes for ferroptosis in psoriasis. Among them, PEBP1, PRKAA2 and ACSF2 are associated with ferroptosis and participate in regulating immune microenvironment in psoriasis cases. Our future studies will focus on the above genes, so as to shed more lights on psoriasis pathogenesis and management. Psoriasis management on the basis of ferroptosis research can be beneficial for psoriatic cases.

## Data availability statement

The datasets presented in this study can be found in online repositories. The names of the repository/repositories and accession number(s) can be found in the article/**Supplementary Materials**.

## Author contributions

M-NW: Data curation; Formal analysis; Methodology; Software; Writing – original draft, C-YJ: Data curation; Writing – review and editing. D-MZ: Methodology; Writing – review and editing. J-CC: Data curation; Writing – review and editing. Y-MZ: Methodology; Writing – review and editing. W-WC: Investigation; Writing – review and editing.TH: Investigation; Writing – review and editing. L-J-MZ: Investigation; Writing – review and editing. All authors contributed to the article and approved the submitted version.

## Supplementary material

Supplementary Figure 1 Single-gene GSEA-KEGG pathway analysis in the marker genes

Supplementary Figure 2 High- and low-expression groups based on the expression levels of each marker gene combined with GSVA.
