# Supplementary figures and images for: Genetic analysis of potential biomarkers and therapeutic targets in ferroptosis from psoriasis
Source: Front Immunol. 2023 Jan 4;13:1104462. doi: 10.3389/fimmu.2022.1104462 (PMC9846571; doi:10.3389/fimmu.2022.1104462)

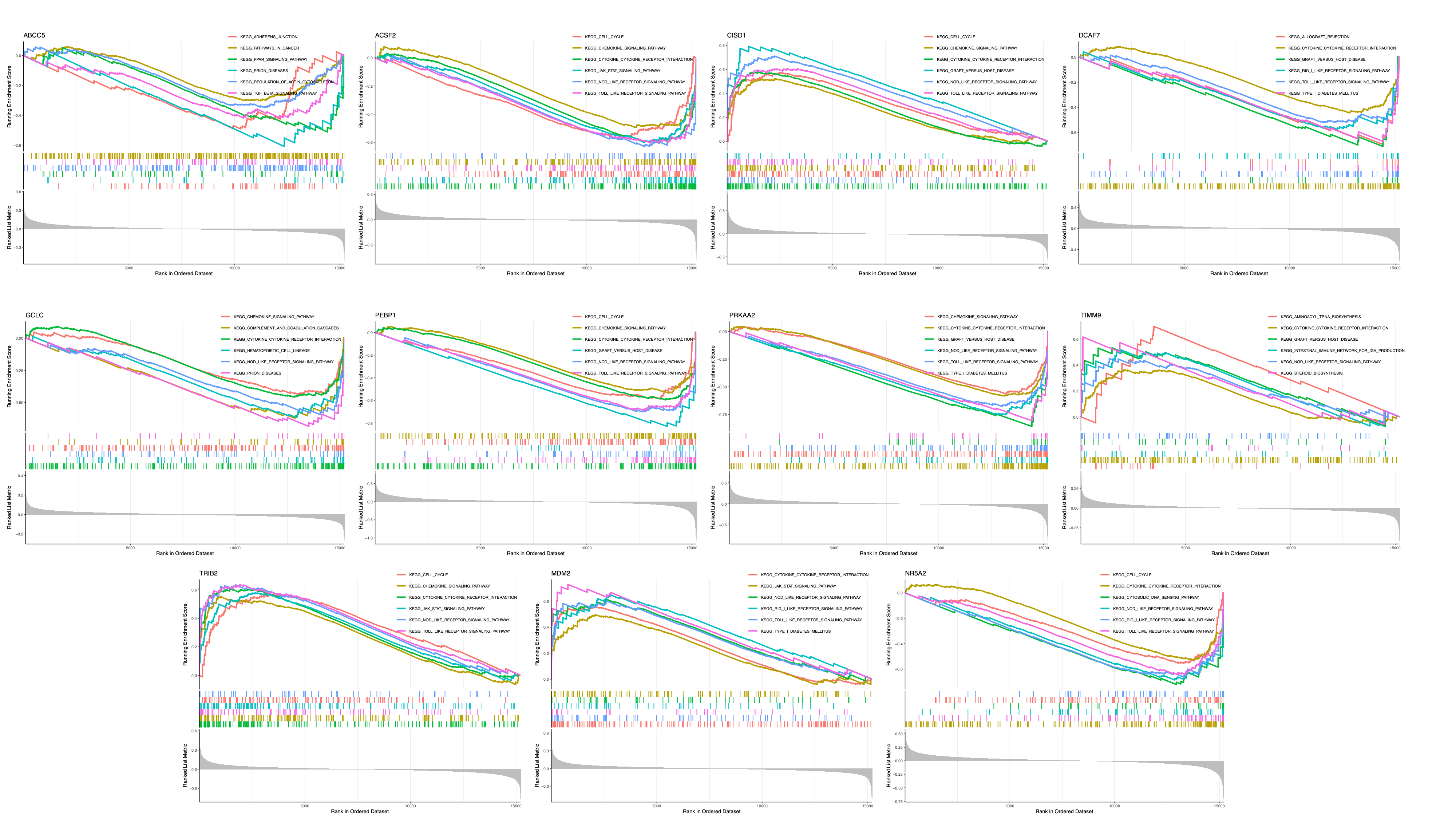

Supplement: Supplementary Figure 1 — Single-gene GSEA-KEGG pathway analysis in the marker genes [file Image_1.tiff]

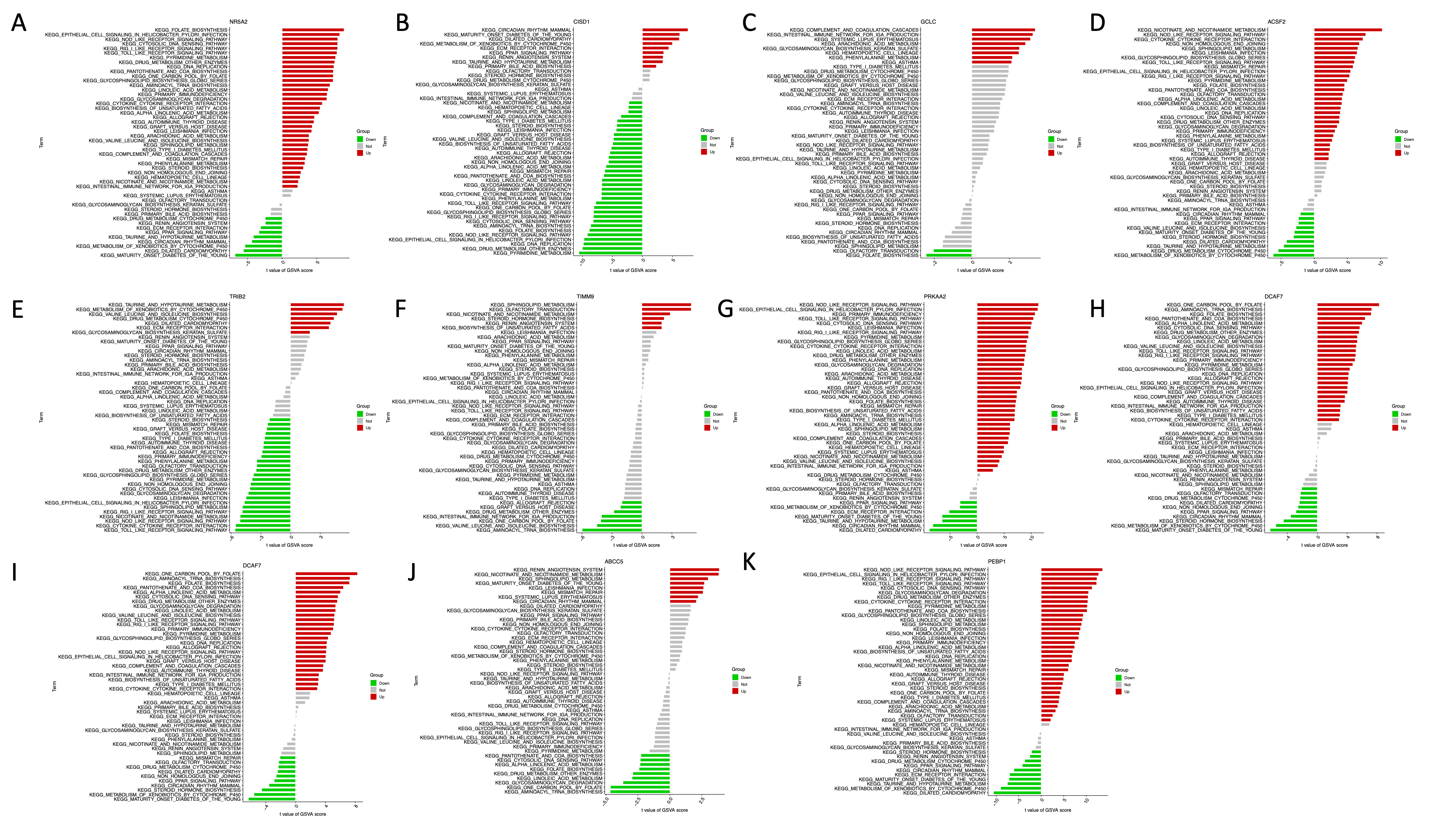

Supplement: Supplementary Figure 2 — High- and low-expression groups based on the expression levels of each marker gene combined with GSVA. [file Image_2.tiff]
